# Role of Elicitors in Inducing Resistance in Plants against Pathogen Infection: A Review

**DOI:** 10.1155/2013/762412

**Published:** 2013-01-28

**Authors:** Meenakshi Thakur, Baldev Singh Sohal

**Affiliations:** Department of Biochemistry, College of Basic Science and Humanities (COBS&H), Punjab Agricultural University, Ludhiana 141 001, India

## Abstract

Disease control is largely based on the use of fungicides, bactericides, and insecticides—chemical compounds toxic to plant invaders, causative agents, or vectors of plant diseases. However, the hazardous effect of these chemicals or their degradation products on the environment and human health strongly necessitates the search for new, harmless means of disease control. There must be some natural phenomenon of induced resistance to protect plants from disease. Elicitors are compounds, which activate chemical defense in plants. Various biosynthetic pathways are activated in treated plants depending on the compound used. Commonly tested chemical elicitors are salicylic acid, methyl salicylate, benzothiadiazole, benzoic acid, chitosan, and so forth which affect production of phenolic compounds and activation of various defense-related enzymes in plants. Their introduction into agricultural practice could minimize the scope of chemical control, thus contributing to the development of sustainable agriculture. This paper chiefly highlights the uses of elicitors aiming to draw sufficient attention of researchers to the frontier research needed in this context.

## 1. Introduction

Plants are challenged by a variety of biotic stresses like fungal, bacterial, or viral infections. This lead to a great loss to plant yield. There are various options available for the farmers to protect their crop from the disease. Some options include development of resistant cultivars, biological control, crop rotation, tillage, and chemical pesticides. Nearly all chemical pesticides or fungicides have a direct antibiotic principle. But their use at commercial level is uneconomical, application is cumbersome, and some are proved to be carcinogenic. Therefore, considerable efforts have been accomplished to devise environmental-friendly strategies for the check of plant diseases and thus to save mankind from health hazard [1].

Plants can activate separate defense pathways depending on the type of pathogen encountered [2]. Jasmonic acid (JA) and ethylene dependent responses seem to be initiated by necrotrophs, whereas salicylic acid (SA) dependent response is activated by biotrophic pathogens. The mechanisms responsible for this differential recognition and response may involve crosstalk among these three different signal transduction pathways: JA, ethylene, and SA.

The better understanding of plant signalling pathways has led to the discovery of natural and synthetic compounds called elicitors that induce similar defense responses in plants as induced by the pathogen infection [3]. Different types of elicitors have been characterized, including carbohydrate polymers, lipids, glycopeptides, and glycoproteins. In plants, a complex array of defense response is induced after detection of microorganism via recognition of elicitor molecules released during plant-pathogen interaction. Following elicitor perception, the activation of signal transduction pathways generally lead to the production of active oxygen species (AOS), phytoalexin biosynthesis, reinforcement of plant cell wall associated with phenyl propanoid compounds, deposition of callose, synthesis of defense enzymes, and the accumulation of pathogenesis-related (PR) proteins, some of which possess antimicrobial properties [4]. AOS lead to hypersensitive response (HR) [5] in plants which is a localized or rapid death of one or few cells at the infection site to delimit the pathogen growth. Following the activation of HR, uninfected distal parts of the plant may develop resistance to further infection, by a phenomenon known as systemic acquired resistance (SAR), which is effective against diverse pathogens, including viruses, bacteria, and fungi [6].

## 2. Host Pathogen Interaction

Resistance in plant species is often divided into host- or nonhost-specific resistance. Host-specific resistance involves interactions between specific host and pathogen genotypes, which give a pathogen race-specific resistance. Nonhost resistance, shown by a whole plant species against a specific parasite or pathogen, is the most common form of resistance in plants towards the majority of potential pathogens [7]. The biochemical changes that occur during infection are very similar in host and nonhost resistant plants [8]. Disease spreads only in susceptible plants (compatible interactions) which are unable to recognize the pathogen or respond too slowly [2]. 

The hypersensitive response is triggered by the plant when it recognizes a pathogen. The identification of a pathogen typically occurs when avirulence (Avr) gene products, secreted by pathogen, bind to or indirectly interact with the product of a plant resistance (R) gene (gene for gene model). When both the R gene and corresponding Avr genes are present, recognition occur, which lead to active resistance of the plant and avirulence of the pathogen. If either Avr gene in the pathogen or R gene in the host is absent or is mutated, no recognition will occur and outcome will be a compatible reaction and disease [9]. As a result of putative binding of these two partners, a signal transduction cascade is activated and lead to the activation of a variety of plant defense responses. The defense responses are associated with restriction of pathogen growth. R gene products are highly polymorphic and many plants produce several different types of R gene products, enabling them to act as a receptor of Avr proteins produced by many different pathogens [7].

### 2.1. Hypersensitive Response (HR)

Direct physiological contact between the host and infecting parasite is obviously necessary for the activation of HR. The HR was first described by Stakman [10] to describe rapid host cell death in resistant wheat plants upon infection by rust fungi. Hypersensitivity is a rapidly developing defense reaction induced in incompatible host by a plant pathogen, which results in the death of a limited number of host cells and a concomitant localization of the pathogen. Some investigators have described the HR as resembling the process of apoptosis, the principal manifestation of programmed cell death in many animal cell types [11]. This definition has now expanded to include defense gene expression in addition to cell death [7]. The HR is analogous to the innate immune response found in animals. HR provides resistance to biotrophic pathogens that obtain their energy from living cells [12].

### 2.2. Generation of Reactive Oxygen Species (ROS)

The first report on the rapid generation of ROS during plant-pathogen interactions was by Doke [13] in *Phytophthora infestans*—potato interaction. In studies involving bacteria and cell suspensions in the incompatible interaction, there are two phases of ROS production, termed as “oxidative burst”. Phase 1 is rapid, transient, and nonspecific, whereas phase 2 occurs later and yields a much higher concentration of ROS [14]. This specific, biphasic response is proposed to be an important component of plant defense [15] because in compatible interactions only the first phase is induced [16]. The two distinct phases of the oxidative burst are seen only when an R gene and an Avr gene are both present, for example, with transgenic tomato plants differing only in the presence or absence of the R gene, *Pto*, and the bacterial pathogen, *Pseudomonas syringae* pv. tomato, with the avr gene, avr*Pto*. This confirms that the second phase of the oxidative burst is associated with disease resistance [17]. The earlier defense responses are the opening of specific ion channels across the plasma membranes, the rapid production of AOS, such as O_2_^−^ and H_2_O_2_, known as the oxidative burst or phosphorylation or dephosphorylation of specific proteins [18]. These initial reactions are the prerequisite for initiation of the signalling network that will trigger the overall defense response [19]. 

### 2.3. Sources of ROS

ROS are toxic intermediates that are generated through the sequential one electron reduction steps of molecular oxygen [20]. Various enzyme systems have been proposed as the source of ROS in plants. An NADPH oxidase system similar to that of mammalian systems or a pH-dependent cell wall peroxidase may be two sources of oxidative burst [21]. If NADPH oxidase activity is a ROS generating system, O_2_^−^ should be the initial product produced, however the O_2_^−^ generated is usually rapidly dismutated to H_2_O_2_ via SOD. Therefore, in most systems H_2_O_2_ appears to be the major ROS that accumulates. Under physiological conditions, the first reduction of O_2_ forms the superoxide anion (O_2_^−^) and hydroperoxyl radical (HO_2_^•^), the second step forms hydrogen peroxide (H_2_O_2_), and the third step produces hydroxyl radical (OH^•^). OH^•^ and O_2_^−^ possess very short half lives. Uncharged H_2_O_2_ is more stable, whereas OH^•^ cannot migrate in solution and instead reacts locally, notably with molecular targets by modifying their structure and activity. H_2_O_2_ as well as OH^•^ can react with polyunsaturated lipids in membranes forming lipid peroxides, which can lead to biological membrane destruction [22].

### 2.4. Role of ROS in Plant Disease Resistance

ROS species such as O_2_^−^, OH^•^, and H_2_O_2_ are commonly produced under stress conditions and are strong oxidizing species that can rapidly attack all types of biomolecules and damage. For the protection from oxidative damage, plant cells contain both oxygen radical detoxifying enzymes such as catalase, peroxidase, and superoxide dismutase, and nonenzymatic antioxidants such as ascorbate peroxidase and glutathione-S-transferase [55]. These enzymes play a crucial role in the protection of plant cells from oxidative damage at the sites of enhanced ROS generation [56]. The cooperative function of these antioxidants plays an important role in scavenging ROS and maintaining the physiological redox status of organisms [57].

### 2.5. Systemic Acquired Resistance (SAR)

Host plants can be protected against further pathogen attack if they have survived earlier infection by phytopathogenic viruses, bacteria, or fungi. It appears that the first infecting pathogen immunizes the plant against further infections by homologous pathogens, even though the plant may not carry gene determining cultivar-specific resistance. The readiness of the plant to repel subsequent pathogen attacks spread throughout the whole plant. This response is called systemic acquired resistance (SAR). The development of SAR is often associated with various cellular defense responses, such as synthesis of PR proteins, phytoalexins and accumulation of AOS, rapid alterations in cell wall, and enhanced activity of various defense related enzymes [58]. 

### 2.6. Sequence of Events Associated with the Establishment of SAR

The onset of SAR in noninfected plant organs is triggered by the phloem mobile signal which is released following pathogen infection. The signal travels throughout the plant and transduced in target tissues. Following signal transduction, resistance is maintained for several days and weeks and this is likely due to de novo gene expression. The biochemical changes that occur during SAR can be divided into two phases, that is, initiation and maintenance. Physiological changes during initiation phase may be transient and short lived, but during maintenance a quasisteady state should exist. 

## 3. Elicitors and Their Mode of Action

Originally the term elicitor was used for molecules capable of inducing the production of phytoalexins, but it is now commonly used for compounds stimulating any type of plant defense [59–61]. Eventually, the induction of defense responses may lead to enhanced resistance. This broader definition of elicitors includes both substances of pathogen origin (exogenous elicitors) and compounds released from plants by the action of the pathogen (endogenous elicitors) [59, 62]. Elicitors are classified as physical or chemical, biotic or abiotic, and complex or defined depending on their origin and molecular structure (Table 1). 

Elicitors may be divided into two groups, “general elicitors” and “race specific elicitors”. While general elicitors are able to trigger defense both in host and nonhost plants, race specific elicitors induce defense responses leading to disease resistance only in specific host cultivars. A complementary pair of genes in a particular pathogen race and a host cultivar determines this cultivar specific (gene-for-gene) resistance. Thus, a race specific elicitor encoded by or produced by the action of an avirulence gene present in a particular race of a pathogen will elicit resistance only in a host plant variety carrying the corresponding resistance gene. The absence of either gene product will often result in disease [19, 63–67]. In contrast, general elicitors signal the presence of potential pathogens to both host and nonhost plants [61]. The nonspecific nature of general elicitors is relative, however, and some of these are only recognized by a restricted number of plants [68]. 

**Figure 1:**
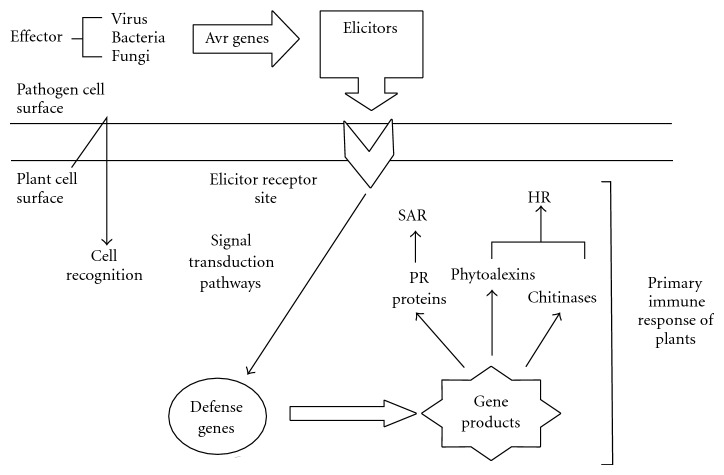
Primary immune response of plant in plant-pathogen interaction.

Recent studies have indicated remarkable similarities between the defense mechanisms triggered by general elicitors and the innate immunity of animals, and it is tempting to speculate that the recognition of general elicitors subsequently leads to plant innate immunity [69]. Elicitors act as signal compounds at low concentrations, providing information for the plant to trigger defense, distinguishing elicitors from toxins, which may act only at higher concentrations and/or affect the plant detrimentally without active plant metabolism [62]. Elicitor signal transduction mechanism which activates plant primary immune response is shown in Figure 1.

## 4. Commercialization

Alternatives to fungicides in plant protection have arisen with the discovery of disease resistance inducers of biotic and abiotic origins that induce a localized or systemic resistance in susceptible plants, which become resistant to subsequent infections. Depending on their efficacy, these compounds can be used in fields either alone or in combination with fungicides. 

Many compounds have been commercially released in some countries as a plant health promoter of annual crops under the name Bion or Actigard [70]. The SA-dependent defense pathway can be activated by treatment of plants with chemical inducers such as benzo (1,2,3)-thiadiazole-7-carbothioic acid-S-methyl ester (acibenzolar-S-methyl, ASM or BTH, Bion) developed as a potent SAR activators which do not only possess antimicrobial properties, but instead increase the crop resistance to diseases by activating SAR signal transduction pathways in several plant species. BTH is a chemical analogue of SA and has been used successfully to induce resistance to a wide range of diseases on field crops. The nonprotein amino acid *β*-aminobutyric acid (BABA) protects numerous plants against various pathogens. Several products have also been used as inducers of resistance in plants against pathogens, including chitosan [71, 72], salicylic acid analogues [24, 73, 74], living or processed fungal products [75], and seaweed extracts [76]. Certain synthetic compounds with no direct antimicrobial effect such as 2,6-dichloroisonicotinic acid (INA) and potassium salts has been reported to induce SAR in plants [77].  Table 2 shows the list of various elicitors used and their effects on different plant species.

## 5. Conclusion

The use of elicitors in crop protection and pest management is still in the very early stages of use as a new control method, and thus the current experiences come from experimental trials, and not yet from large scale agricultural use. At least the following advantages of using elicitor treatments have been reported or can be expected:

1. reduced damage from insects, fungi, pests, and herbivores,
2. reduced environmental hazards as elicitors affect directly the crop plant, and their acute toxicity to other organisms is lower than that of pesticides,
3. as protective agrochemicals, elicitors can be applied with the current spraying technology,
4. elicitor treatments could be an alternative to genetically modified (GM) plants for better attraction of natural enemies of pest organisms on cultivated plants [78],
5. elicitor-treated plants bear lower ecological risks than GM plants [79].

## Figures and Tables

**Table 1:** List of various types of plant elicitors.

Type of elicitors and their examples			
*Physical elicitors*	Injury		
*Chemical elicitors*	*Abiotic elicitors*: Metal ions		
	*Biotic elicitors: *		
	(1) Complex composition	(2) Defined composition	
	Yeast cell wall, mycelia cell wall, and fungal spores	(2.1) Carbohydrates	
		Polysaccharides:	Oligosaccharides:
		Alginate, pectin, and chitosan	Mannuronate, guluronate, mannan, and galacturonides
		(2.2) Proteins	
		Peptides:	Proteins:
		Glutathione	Cellulase and oligandrin
		(2.3) Lipids	
		Lipopolysaccharides	
		(2.4) Glycoproteins	
		Not characterized	
		(2.5) Volatiles	
		C_6_–C_10 _compounds	

**Table 2:** List of elicitors used and their effects on different plant species.

S. No.	Plant	Type of elicitor used	Effects	References

1	*Brassica napus *	Methyl jasmonate	Accumulation of indolyl glucosinolates in the leaves. The predominant components of the response were 3-indolylmethyl- and 1-methoxy-3-indolylmethylglucosinolates, which together comprised 90% of the total glucosinolates in treated leaves.	[23]

2	*Oryza sativa *	Benzothiadiazole	BTH protected wheat systemically against powdery mildew infection by affecting multiple steps in the life cycle of the pathogen. The onset of resistance was accompanied by the induction of a number of wheat chemically induced (WCI) genes, including genes encoding a lipoxygenase and a sulfur-rich protein.	[24]

3	*Lycopersicon esculentum *	Salicylic acid	Diminished susceptibleness to pathogens harm and abiotic stress.	[25]

4	*Beta vulgaris *	Benzothiadiazole	Induced synthesis of chitinase and *β*-1,3-glucanase isozymes providing resistance against tobacco necrosis virus.	[26]

5	*Brassica oleracea *(var.* Botrytis*)	Benzothiadiazole	BTH induced downy mildew (caused by *P*. *parasitica*) resistance in both cauliflower seedlings and 30-day old plants.	[27]

6	*Lycopersicon esculentum, Commelina communis *	Oligogalacturonic acid (OGA) and chitosan	These elicitors reduced the size of the stomatal aperture. OGA not only inhibited light-induced stomatal opening, but also accelerated stomatal closing in both species; chitosan inhibited light-induced stomatal opening in tomato epidermis.	[28]

7	*Musa acuminata *	Salicylic acid	Delayed ripening of banana fruit.	[29]

8	*Lycopersicon esculentum *	Salicylic acid	Induced the synthesis of some stress proteins, such as PR proteins, which leads to increased chilling tolerance and resistance to pathogens, thereby decreasing the incidence of decay.	[30]

9	*Lilium *	Benzoic acid	Modified the growth, stress tolerance, anatomy and morphology of eatable and ornamental species.	[31]

10	*Helianthus annuus *	Benzothiadiazole	Prevented infestation by the parasitic weed *Orobanche cumana*. Root exudates revealed synthesis of the phytoalexin scopoletin, PR-protein chitinase and H_2_O_2_.	[32]

11	*Avena sativa, Oryza sativa, Raphanus sativus, Arachis hypogea, Nicotiana tabacum, Pisum sativum*	Chitosan	Act as a stress tolerance inductor when directly applied to plant tissues, unchaining a hypersensitive reaction and lignifications, and promoting the activation of defenses against pathogens.	[33]

12	*Lycopersicum esculentum *	Chitosan	Produced a higher resistance against *Fusarium oxysporum* and *Phytophthora capsici. *	[34]

13	*Lycopersicon esculentum *(var*. Castlemart*)	Salicylic acid	Upregulation of transcription of PR1 and BGL2 genes (marker genes of SA pathway), increased endogenous H_2_O_2_ level involved in resistance against *Helicoverpa armigera. *	[35]

14	*Pisum sativum *	Salicylic acid and 4-aminobutyric acid	Increased activity of phenol metabolizing enzymes *viz*., POD, PPO, PAL providing resistance against* Erysiphe. polygony* in pea.	[36]

15	*Brassica juncea *	Benzothiadiazole	Increased phenolics and extracellular proteins act as markers of induced resistance.	[37]

16	*Lycopersicon esculentum *	Chitosan and salicylic acid	Increased level of catalase and peroxidase enzymes activity.	[38]

17	*Citrus sinensis *	*β*-amino butyric acid	Inhibited *Penicillium italicum* spore germination and germ tube elongation *in vitro.* Involved in the induced resistance against *Penicillium italicum. *	[39]

18	*Glycine max *	Benzothiadiazole	Decreased incidence of soybean stem vascular discoloration, increased germination, photosynthetic pigments, lignin, phenolics, and flavonoids. Increased activities of phenylalanine ammonia lyase, peroxidase, and polyphenoloxidase. Decreased catalase activity was observed.	[40]

19	Bhendi	Salicylic acid	Accumulation of phenolics and increased activity of enzyme PAL leading to resistance against *Erysiphe cichoracearum. *	[41]

20	*Brassica* species	Salicylic acid	Recovery from heat stress, increased seedling length, reduced electrolyte leakage, and enhanced membrane protection. Increased level of total soluble sugars, fresh/dry weight, and enzymatic activities of invertase, catalase, and peroxidase conferred thermotolerance. Enhanced expression of some new proteins including heat shock proteins (HSPs) was also observed.	[42]

21	*Brassica napus *	Salicylic acid and nitric oxide	Increased the activities of the antioxidant enzymes in leaves of Ni-stressed plants, improved the chlorophyll content and decreased the level of lipid peroxidation, and H_2_O_2 _and proline accumulation in leaves.	[43]

22	*Solanum melongena *	Salicylic acid, chitosan, methyl salicylate, and methyl jasmonate	Increased lignin deposition in cell walls of roots, accumulation of phenolics, increase in the activity of enzymes PAL, POD, polyphenol oxidase, cinnamyl alcohol dehydrogenase, and catalase. Provided resistance against *Ralstonia solanacearum. *	[44]

23	*Brassica juncea* (var. Rlm619)	Benzothiadiazole and salicylic acid	Induction of defense related enzymes, namely, peroxidase, phenylalanine ammonia lyase, and superoxide dismutase; increase in oil content and yield. Prevention of invasion of *Alternaria brassicae*.	[45]

24	*Phaseolus vulgaris *	Salicylic acid and Methyl jasmonate	Controlled spider mite infestation, improved plant growth and bean yield.	[46]

25	*Brassica oleracea *(var. *Italica*)	Methionine, tryptophan, chitosan, salicylic acid, and methyl jasmonate	Salicylic acid and chitosan induced increase in vitamin C content. Flavonoid concentration increased after MeJA and SA treatments. Methionine or tryptophan solutions did not positively affect the vitamin C or the phenolic compounds. Methionine increased the levels of aliphatic glucosinolates. However, indole glucosinolates presented a significant response to the induction with tryptophan, SA, or MeJA treatments.	[47]

26	*Glycine max *	Benzothiadiazole and humic acid	Reduced damping-off and wilt diseases and increased growth parameters. BTH and HA in combination showed the highest activities of oxidative enzymes followed by BTH and HA alone.	[48]

27	Soybean, rice, and wheat	*β*-glucans and chitin oligomers from *Phytophthora* and *Pythium *	Produced phytoalexins in soybean and rice plants. Lignification in wheat leaves.	[49]

28	*Arabidopsis, tomato *	Oligogalacturonides from bacteria and fungi	Synthesis of protein inhibitors and activation of defense genes.	[50]

29	Tobacco, tomato	Viral coat protein harpin from TMV	Activation of hypersensitive response.	[49]

30	Tomato	Avr gene products, for example, AVR4 and AVR9 from *Cladosporium fulvum *	Activation of hypersensitive response.	[51]

31	*Arabidopsis *	Flagellin, flg 15 from gram negative bacteria	Deposition of callose and activation of defense genes in *Arabidopsis*.	[52]

32	Oat	Protein or peptide toxin, victorin from *Helminthosporium victoriae* (rust)	Programmed cell death in oat.	[53]

33	Parsley	Glycoprotein from *Phytophthora sojae *	Synthesis of phytoalexin and activation of defense genes in parsley.	[49]

34	Soybean	Syringolids from *Pseudomonas syringae *	Activation of hypersensitive response.	[49]

35	Tobacco	Fatty acid amino acid conjugates from Lepidopterans	Synthesis of monoterpenes leading to activation of indirect defense in tobacco.	[49]

36	*Arabidopsis *	Bacterial toxin, for example, coronatine from *Pseudomonas syringae *	Acivation of defense genes in *Arabidopsis*.	[54]

37	*Arabidopsis, tomato *	Sphinganine analogue mycotoxins from *Fusarium moniliforme *	Programmed cell death and activation of defense genes in *Arabidopsis* and tomato.	[49]

## References

[1] El-Gamal N. G., Abd-El-Kareem F., Fotouh Y. O., El Mougy N. S. (2007). Induction of systemic resistance in potato plants against late and early blight diseases using chemical inducers under greenhouse and field conditions. *Research Journal of Agriculture and Biological Sciences*.

[2] Garcia-Brugger A., Lamotte O., Vandelle E. (2006). Early signaling events induced by elicitors of plant defenses. *Molecular Plant-Microbe Interactions*.

[3] Gómez-Vásquez R., Day R., Buschmann H., Randles S., Beeching J. R., Cooper R. M. (2004). Phenylpropanoids, phenylalanine ammonia lyase and peroxidases in elicitor-challenged cassava (*Manihot esculenta*) suspension cells and leaves. *Annals of Botany*.

[4] Van Loon L. C., Van Strien E. A. (1999). The families of pathogenesis-related proteins, their activities, and comparative analysis of PR-1 type proteins. *Physiological and Molecular Plant Pathology*.

[5] Agrios G. N. (1988). *Plant Pathology*.

[6] Heil M., Bostock R. M. (2002). Induced systemic resistance (ISR) against pathogens in the context of induced plant defences. *Annals of Botany*.

[7] Heath M. C. (2000). Hypersensitive response-related death. *Plant Molecular Biology*.

[8] Somssica I. E., Hahlbrock K. (1998). Pathogen defence in plants—a paradigm of biological complexity. *Trends in Plant Science*.

[9] De Wit P. J. G. M. (1995). Fungal avirulence genes and plant resistance genes: unraveling the molecular basis of gene-for-gene interactions. *Advances in Botanical Research*.

[10] Stakman E. C. (1915). Relation between *Puccinia graminis* and plants highly resistant to its attack. *Agricultural Research*.

[11] Morel J. B., Dangl J. L. (1997). The hypersensitive response and the induction of cell death in plants. *Cell Death and Differentiation*.

[12] Kumar J., Hückelhoven R., Beckhove U., Nagarajan S., Kogel K. H. (2001). A compromised Mlo pathway affects the response of barley to the necrotrophic fungus *Bipolaris sorokiniana* (teleomorph: *Cochliobolus sativus*) and its toxins. *Phytopathology*.

[13] Doke N. (1983). Involvement of superoxide anion generation in the hypersensitive response of potato tuber tissues to infection with an incompatible race of *Phytophthora infestans* and to the hyphal wall components. *Physiological Plant Pathology*.

[14] Baker C. J., O'Neill N. R., Keepler L. D., Orlandi E. W. (1991). Early responses during plant- bacteria interactions in tobacoo cell suspensions. *Phytopathology*.

[15] Lamb C., Dixon R. A. (1997). The oxidative burst in plant disease resistance. *Annual Review of Plant Physiology and Plant Molecular Biology*.

[16] Levine A., Tenhaken R., Dixon R., Lamb C. (1994). H_2_O_2_ from the oxidative burst orchestrates the plant hypersensitive disease resistance response. *Cell*.

[17] Chandra S., Martin G. B., Low P. S. (1996). The Pto kinase mediates a signaling pathway leading to the oxidative burst in tomato. *Proceedings of the National Academy of Sciences of the United States of America*.

[18] Conrath U., Silva H., Klessig D. F. (1997). Protein dephosphorylation mediates salicylic acid-induced expression of PR-1 genes in tobacco. *Plant Journal*.

[19] Hammond-Kosack K. E., Jones J. D. G. (1996). Resistance gene-dependent plant defense responses. *The Plant Cell*.

[20] Mehdy M. C. (1994). Active oxygen species in plant defense against pathogens. *Plant Physiology*.

[21] Wojtaszek P. (1997). Oxidative burst: an early plant response to pathogen infection. *Biochemical Journal*.

[22] Grant J. J., Loake G. J. (2000). Role of reactive oxygen intermediates and cognate redox signaling in disease resistance. *Plant Physiology*.

[23] Doughty K. J., Kiddle G. A., Pye B. J., Wallsgrove R. M., Pickett J. A. (1995). Selective induction of glucosinolates in oilseed rape leaves by methyl jasmonate. *Phytochemistry*.

[24] Görlach J., Volrath S., Knauf-Beiter G. (1996). Benzothiadiazole, a novel class of inducers of systemic acquired resistance, activates gene expression and disease resistance in wheat. *The Plant Cell*.

[25] Shirasu K., Nakajima H., Rajasekhar V. K., Dixon R. A., Lamb C. (1997). Salicylic acid potentiates an agonist-dependent gain control that amplifies pathogen signals in the activation of defense mechanisms. *The Plant Cell*.

[26] Burketová L., Šindelářová M., Šindelář L. (1999). Benzothiadiazole as an inducer of *β*-1,3-glucanase and chitinase isozymes in sugar beet. *Biologia Plantarum*.

[27] Godard J. F., Ziadi S., Monot C., Le Corre D., Silué D. (1999). Benzothiadiazole (BTH) induces resistance in cauliflower (*Brassica oleracea* var botrytis) to downy mildew of crucifers caused by *Peronospora parasitica*. *Crop Protection*.

[28] Lee S., Choi H., Suh S. (1999). Oligogalacturonic acid and chitosan reduce stomatal aperture by inducing the evolution of reactive oxygen species from guard cells of tomato and *Commelina communis*. *Plant Physiology*.

[29] Srivastava M. K., Dwivedi U. N. (2000). Delayed ripening of banana fruit by salicylic acid. *Plant Science*.

[30] Garcia-Magallon E., Rojas-Duarte A., Benavides-Mendoza A., Ramírez-Godina F., Bañuelos-Herrera L. (2002). Aplicación del ácido benzoico en forma foliar al cultivo de *Lilium* cv. Dreamland. *Memoria del XIX Congreso Nacional de Fitogenética*.

[31] Ding C. K., Wang C. Y., Gross K. C., Smith D. L. (2002). Jasmonate and salicylate induce the expression of pathogenesis-related-protein genes and increase resistance to chilling injury in tomato fruit. *Planta*.

[32] Sauerborn J., Buschmann H., Ghiasi K. G., Kogel K. H. (2002). Benzothiadiazole activates resistance in sunflower (*Helianthus annuus*) to the root-parasitic weed *Orobanche cumana*. *Phytopathology*.

[33] Maksimov I. V., Cherepanova E. A., Khairullin R. M. (2003). ‘Chitin-specific’ peroxidases in plants. *Biochemistry*.

[34] Ortega-Ortíz H., Benavides-Mendoza A., Flores-Olivas A., Ledezma-Pérez A. (2003). Use of the interpolyelectrolyte complexes of poly(acrylic acid)-chitosan as inductors of tolerance against pathogenic fungi in tomato (*Lycopersicon esculentum* Mill. var. Floradade). *Macromolecular Bioscience*.

[35] Peng J., Deng X., Huang J., Jia S., Miao X., Huang Y. (2004). Role of salicylic acid in tomato defense against cotton bollworm, *Helicoverpa armigera* Hubner. *Zeitschrift fur Naturforschung*.

[36] Katoch R. (2005). Effect of elicitors and *E. polygoni* inoculation on the activity of phenol metabolizing enzymes in garden pea (*Pisum sativum* L.). *Indian Journal of Agricultural Biochemistry*.

[37] Guleria S., Kumar A. (2006). Qualitative profiling of phenols and extracellular proteins induced in mustard (*Brassica juncea*) in response to benzothiadiazole treatment. *Journal of Cell Molecular Biology*.

[38] Ortega-Ortiz H., Benavides-Mendoza A., Mendoza-Villarreal R., Ramirez-Rodriguez H., Romenus K. D. A. (2007). Enzymatic activity in tomato fruits as a response to chemical elicitors. *Journal of Mexican Chemical Society*.

[39] Tavallali V., Karimi S., Mohammadi S., Hojati S. (2008). Effects of *β*-aminobutyric acid on the induction of resistance to *Penicillium italicum*. *World Applied Science Journal*.

[40] Nafie E., Mazen M. M. (2008). Chemical-induced resistance against brown stem rot in soybean: the effect of benzothiadiazole. *Journal of Applied Science Research*.

[41] Vimala R., Suriachandraselvan M. (2009). Induced resistance in bhendi against powdery mildew by foliar application of salicylic acid. *Journal of Biopesticides*.

[42] Kaur P., Ghai N., Sangha M. K. (2009). Induction of thermotolerance through heat acclimation and salicylic acid in *Brassica* species. *African Journal of Biotechnology*.

[43] Kazemi N., Khavari-Nejad R. A., Fahimi H., Saadatmand S., Nejad-Sattari T. (2010). Effects of exogenous salicylic acid and nitric oxide on lipid peroxidation and antioxidant enzyme activities in leaves of *Brassica napus* L. under nickel stress. *Scientia Horticulturae*.

[44] Mandal S. (2010). Induction of phenolics, lignin and key defense enzymes in eggplant (*Solanum melongena* L.) roots in response to elicitors. *African Journal of Biotechnology*.

[45] Sharma S., Sohal B. S. (2010). Foliar spray of benzothiadiazole and salicylic acid on *Brassica juncea* var. Rlm619 to combat *Alternaria* blight in field trials. *Crop Improvement*.

[46] Farouk S., Osman M. A. (2011). The effect of plant defense elicitors on common bean (*Phaseolus vulgaris* L.) growth and yield in absence or presence of spider mite (*Tetranychus urticae* Koch) infestation. *Journal of Stress Physiology and Biochemistry*.

[47] Pérez-Balibrea S., Moreno D. A., García-Viguera C. (2011). Improving the phytochemical composition of broccoli sprouts by elicitation. *Food Chemistry*.

[48] Abdel-Monaim M. F., Ismail M. E., Morsy K. M. (2011). Induction of systematic resistance in soybean plants against *Fusarium* wilt disease by seed treatment with benzothiadiazole and humic acid. *Notulae Scientia Biologicae*.

[49] Montesano M., Brader G., Palva E. T. (2003). Pathogen derived elicitors: searching for receptors in plants. *Molecular Plant Pathology*.

[50] Shibuya N., Minami E. (2001). Oligosaccharide signalling for defence responses in plant. *Physiological and Molecular Plant Pathology*.

[51] Leach J. E., White F. F. (1996). Bacterial avirulence genes. *Annual Review of Phytopathology*.

[52] Gómez-Gómez L., Boller T. (2000). FLS2: an LRR receptor-like kinase involved in the perception of the bacterial elicitor flagellin in *Arabidopsis*. *Molecular Cell*.

[53] Tada Y., Hata S., Takata Y., Nakayashiki H., Tosa Y., Mayama S. (2001). Induction and signaling of an apoptotic response typified by DNA laddering in the defense response of oats to infection and elicitors. *Molecular Plant-Microbe Interactions*.

[54] Kloek A. P., Verbsky M. L., Sharma S. B. (2001). Resistance to *Pseudomonas syringae* conferred by an *Arabidopsis thaliana* coronatine-insensitive (coi1) mutation occurs through two distinct mechanisms. *Plant Journal*.

[55] Pnueli L., Liang H., Rozenberg M., Mittler R. (2003). Growth suppression, altered stomatal responses, and augmented induction of heat shock proteins in cytosolic ascorbate peroxidase (Apx1)-deficient *Arabidopsis* plants. *Plant Journal*.

[56] Kuniak E., Sklodowska M. (2001). Ascorbate, glutathione and related enzymes in chloroplasts of tomato leaves infected by *Botrytis cinerea*. *Plant Science*.

[57] Cho U. H., Seo N. H. (2005). Oxidative stress in *Arabidopsis thaliana* exposed to cadmium is due to hydrogen peroxide accumulation. *Plant Science*.

[58] Ryals J. A., Neuenschwander U. H., Willits M. G., Molina A., Steiner H. Y., Hunt M. D. (1996). Systemic acquired resistance. *The Plant Cell*.

[59] Ebel J., Cosio E. G. (1994). Elicitors of plant defense responses. *International Review of Cytology*.

[60] Hahn M. G. (1996). Microbial elicitors and their receptors in plants. *Annual Review of Phytopathology*.

[61] Nürnberger T. (1999). Signal perception in plant pathogen defense. *Cellular and Molecular Life Science*.

[62] Boller T. (1995). Chemoperception of microbial signals in plant cells. *Annual Review of Plant Physiology and Plant Molecular Biology*.

[63] Cohn J., Sessa G., Martin G. B. (2001). Innate immunity in plants. *Current Opinion in Immunology*.

[64] Luderer R., Joosten M. H. A. J. (2001). Avirulence proteins of plant pathogens: determinants of victory and defeat. *Molecular Plant Pathology*.

[65] Nimchuk Z., Rohmer L., Chang J. H., Dangl J. L. (2001). Knowing the dancer from the dance: R-gene products and their interactions with other proteins from host and pathogen. *Current Opinion in Plant Biology*.

[66] Nürnberger T., Scheel D. (2001). Signal transmission in the plant immune response. *Trends in Plant Science*.

[67] Tyler B. M. (2002). Molecular basis of recognition between Phytophthora pathogens and their hosts. *Annual Review of Phytopathology*.

[68] Shibuya N., Minami E. (2001). Oligosaccharide signalling for defence responses in plant. *Physiological and Molecular Plant Pathology*.

[69] Nürnberger T., Brunner F. (2002). Innate immunity in plants and animals: emerging parallels between the recognition of general elicitors and pathogen-associated molecular patterns. *Current Opinion in Plant Biology*.

[70] Chen P., Li Z. (2006). BTH systemic induction to defense related enzymes in wheat leaves. *Acta Botanica Boreali-Occidentalia Sinica*.

[71] Bohland C., Balkenhohl T., Loers G., Feussner I., Grambow H. J. (1997). Differential induction of lipoxygenase isoforms in wheat upon treatment with rust fungus elicitor, chitin oligosaccharides, chitosan, and methyl jasmonate. *Plant Physiology*.

[72] Reddy M. V. B., Arul J., Angers P., Couture L. (1999). Chitosan treatment of wheat seeds induces resistance to *Fusarium graminearum* and improves seed quality. *Journal of Agricultural and Food Chemistry*.

[73] Benhamou N., Bélanger R. R. (1998). Induction of systemic resistance to *Pythium* damping-off in cucumber plants by benzothiadiazole: ultrastructure and cytochemistry of the host response. *Plant Journal*.

[74] Brisset M. N., Cesbron S., Thomson S. V., Paulin J. P. (2000). Acibenzolar-S-methyl induces the accumulation of defense-related enzymes in apple and protects from fire blight. *European Journal of Plant Pathology*.

[75] Hjeljord L. G., Stensvand A., Tronsmo A. (2000). Effect of temperature and nutrient stress on the capacity of commercial *Trichoderma* products to control *Botrytis cinerea* and *Mucor piriformis* in greenhouse strawberries. *Biological Control*.

[76] Washington W. S., Engleitner S., Boontjes G., Shanmuganathan N. (1999). Effect of fungicides, seaweed extracts, tea tree oil, and fungal agents on fruit rot and yield in strawberry. *Australian Journal of Experimental Agriculture*.

[77] Oostendorp M., Kunz W., Dietrich B., Staub T. (2001). Induced disease resistance in plants by chemicals. *European Journal of Plant Pathology*.

[78] Kappers I. F., Aharoni A., van Herpen T. W. J. M., Luckerhoff L. L. P., Dicke M., Bouwmeester H. J. (2005). Genetic engineering of terpenoid metabolism attracts bodyguards to *Arabidopsis*. *Science*.

[79] Poppy G. M., Wilkinson M. J. (2005). *Gene Flow from GM Plants—A Manual for Assessing, Measuring and Managing the Risks*.

